# Two-stage aortic surgery for distal aortic arch and descending aorta aneurysms: A case report

**DOI:** 10.1097/MD.0000000000030342

**Published:** 2022-09-09

**Authors:** Akie Shimada, Taira Yamamoto, Shizuyuki Dohi, Yasutaka Yokoyama, Daisuke Endo, Minoru Tabata

**Affiliations:** a Department of Cardiovascular Surgery, Nerima Hospital, Juntendo University, Nerima-ku, Tokyo, Japan; b Department of Cardiovascular Surgery, Juntendo University, Bunkyo-ku, Tokyo, Japan.

**Keywords:** aortic regurgitation, atrial septal defect, cerebral aneurysm, distal aortic arch aneurysm, hemodialysis

## Abstract

Although surgical treatment strategies for patients with extensive thoracic aortic disease involving the aortic arch have improved considerably, the impact of stent graft length and placement site on aortic remodeling at long-term follow-up is not fully understood, and the protection of the Adamkiewicz artery (AKA) using the frozen elephant trunk (FET) method is also unclear.

**Patient concerns:**

The patient was a 69-year-old man with diabetic nephropathy who became increasingly fatigued and started maintenance hemodialysis 6 months prior to admission. At 64 years, he underwent clipping of a right cerebellar artery aneurysm. In addition, a 1.8 cm aneurysm was found in the contralateral extracranial internal carotid artery. He also had an atrial septal defect and moderate aortic regurgitation and was receiving continuous positive airway pressure therapy for sleep apnoea syndrome.

**Diagnosis:**

He had aneurysms in the aortic arch (4.8 cm in diameter) and descending aorta (6 cm in diameter), which was located at T6–9. Preoperative 3-dimensional computed tomography showed that the (AKA) bifurcated at T10–11.

**Interventions:**

Considering the patient’s several comorbidities and frailty, we planned to perform 1-stage extended aortic arch repair using the FET procedure. However, we performed 2-stage aortic surgery to prevent spinal ischemia, anticipating substantial cardiac enlargement and blood pressure instability due to dialysis treatment. Aortic valve replacement, atrial septal defect patch closure, and aortic arch surgery were performed. A 7-cm elephant trunk was inserted in the descending aorta. Postoperatively, the patient continued rehabilitation until his blood pressure stabilized during dialysis therapy. At postoperative week 4, he underwent thoracic endovascular aortic repair for a descending aortic aneurysm.

**Outcomes:**

After surgery, his physical strength decreased; however, he recovered and was discharged 1 month later without any complications. One year after the second operation, he is living a healthy life.

**Lessons:**

Extensive aortic arch surgery using the FET procedure is effective for distal aortic arch and descending aortic aneurysms. Nevertheless, in cases in which the position of the AKA is close to the aortic aneurysm and blood pressure control is difficult, a 2-stage procedure and accurate positioning of thoracic endovascular aortic repair are both desirable.

## 1. Introduction

Surgical repair of extensive lesions from the aortic arch to the descending aorta often requires complex staged surgery. The management of cerebral circulation during intraoperative circulatory arrest and after reperfusion involves careful handling and poses risks. Over the past 2 decades, surgical treatment strategies for patients with extensive thoracic aortic diseases, including those in the aortic arch region, have considerably improved, which has enabled surgeons to select the optimal approach from several treatment options.^[1,2]^ A particular breakthrough is the introduction of the frozen elephant trunk (FET) technique,^[3]^ which has simplified complex anastomosis and shortened the myocardial and visceral ischemia time by bringing the distal anastomosis closer to zone 2 from zone 3.^[4]^ Nonetheless, the impact of stent graft length and deployment site on aortic remodeling at long-term follow-up has not been fully elucidated, and the protection of the Adamkiewicz artery (AKA) using the FET technique is uncertain.^[5,6]^

We describe the treatment of a 69-year-old male patient with aneurysms in the distal aortic arch and descending aorta undergoing hemodialysis for whom the treatment decision was difficult. He had a history of heart failure and a huge cerebral aneurysm. We initially planned to perform 1-stage aortic arch repair using the FET procedure. However, preoperative examination showed adverse requirements for the FET technique regarding postoperative blood pressure control and the anatomic location of the AKA and descending aortic aneurysm. Therefore, we performed a 2-stage repair and obtained with favorable results.

## 2. Case presentation

A 69-year-old man diagnosed with hypertension and diabetes mellitus at 57 years of age and with impaired renal function due to diabetic nephropathy since the age of 60 years continued to receive management care. His chronic kidney disease gradually worsened, and results from blood sample analysis were as follows: serum creatinine, 6.20 mg/dL; blood urea nitrogen, 67 mg/dL; estimated glomerular filtration rate, 7.85 mL/min/1.73 m^2^. He underwent clipping surgery for a right basilar artery aneurysm at 64 years of age and had been receiving continuous positive airway pressure treatment for sleep apnoea since the age of 60 years. His medications included amlodipine besylate (10 mg/day), telmisartan (40 mg/day), sodium polystyrene sulfonate (10 g/day), febuxostat (30 mg/day), atorvastatin calcium hyaluronate (30 mg/day), and atorvastatin calcium hydrate (10 mg/day). His blood pressure on routine outpatient visits ranged from 130/60 mm Hg to 150/78 mm Hg. During the cardiology examination before the shunt operation for dialysis, distal aortic arch aneurysm, descending aortic aneurysm, aortic regurgitation (AR), and atrial septal defect (ASD) were detected; accordingly, the medical doctor consulted our department. The patient was admitted to our hospital, and dialysis was initiated due to decreased appetite, increased pericardial fluid, and pleural effusion.

Physical examination revealed a height of 170 cm, weight of 65.6 kg, blood pressure of 144/60 mm Hg, and heart rate of 78 bpm with sinus rhythm. He had remarkable oedema in the bilateral lower legs. Physical fitness tests included a 5-m walk in 4.98 seconds, endurance test (210-m walk in 195 seconds), 1-legged stance test (right leg, 4.8 seconds; left leg, 8.9 seconds), grip strength test (right hand, 34 kg; left hand, 21 kg), and Short Physical Performance Battery test (total, 9/12 points; balance, 4 points; walking, 3 points; standing up, 2 points).

The laboratory data were as follows: hemoglobin, 12.8 g/dL; platelet count, 144 × 10^9^/L; total protein, 6.1 mg/dL; albumin, 3.6 mg/dL; total bilirubin, 0.5 mg/dL; aspartate aminotransferase, 19 mg/dL; alanine aminotransferase, 24 IU/L; prothrombin time–international normalized ratio, 0.97; activated partial thromboplastin time, 28.4 seconds; serum creatinine, 7.67 mg/dL; blood urea nitrogen, 60 mg/dL; hemoglobin A1c, 6.2%; N-terminal prohormone of brain natriuretic peptide, 3321 pg/mL; and C-reactive protein, 0.09 mg/L.

**Figure 1.**
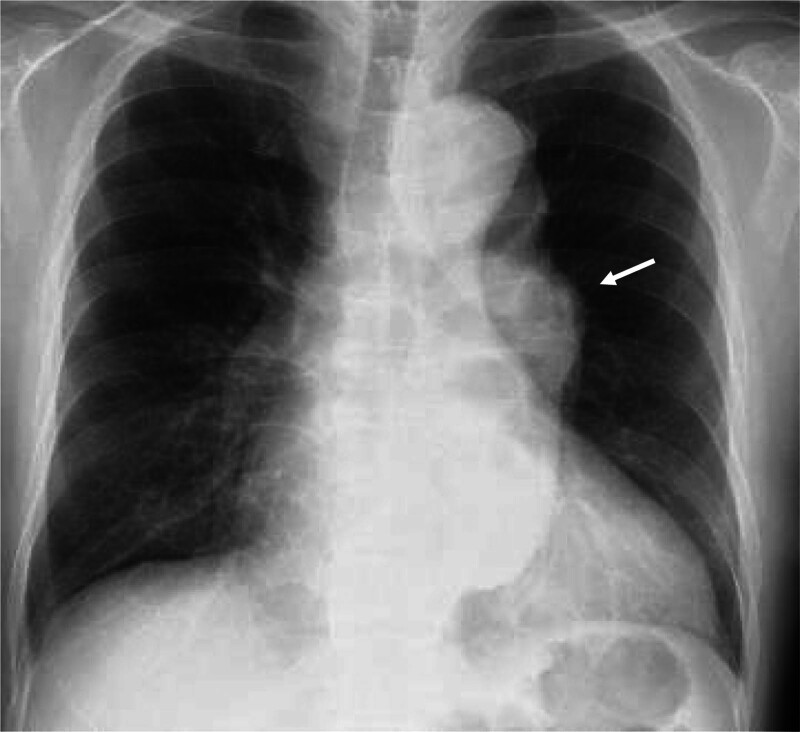
Preoperative chest radiography findings. Chest radiography indicated a cardiothoracic ratio of 65%. The white arrow indicates descending aortic aneurysm.

**Figure 2.**
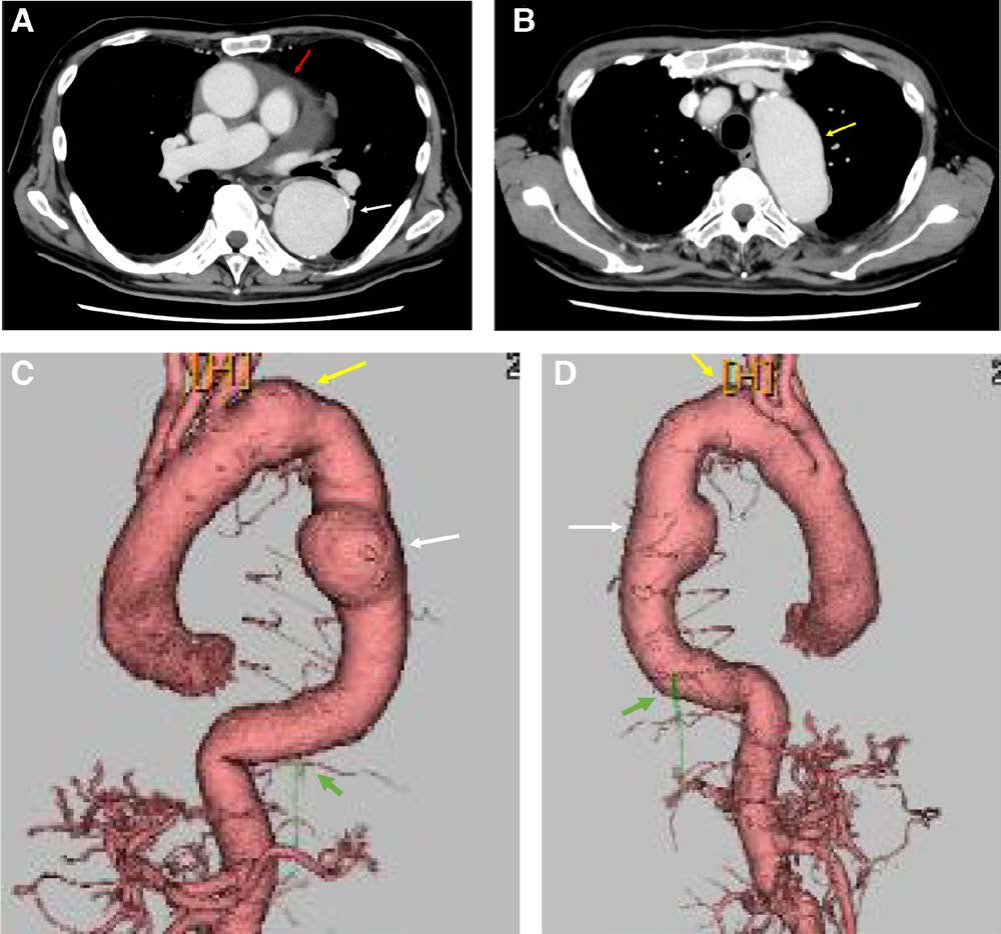
Preoperative computed tomography. (A) The red arrow indicates pericardial effusion, whereas the white arrow indicates descending aortic aneurysm. (B) The yellow arrow indicates distal aortic arch aneurysm. (C and D) The red, yellow, white, and green arrows indicate pericardial effusion, distal aortic arch aneurysm, descending aortic aneurysm, and the Adamkiewicz artery, respectively.

**Figure 3.**
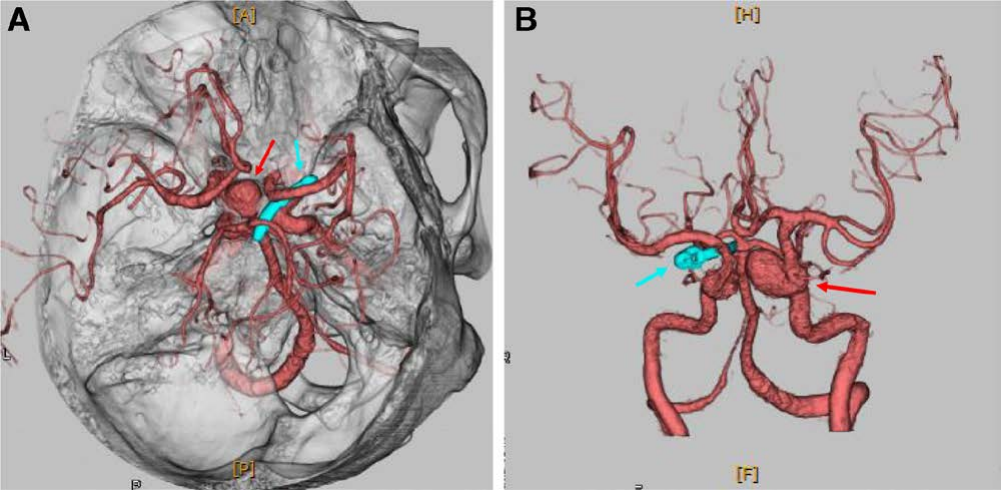
Three-dimensional computed tomography of the brain. The blue arrow indicates the clip for the prior cerebral aneurysm, whereas the red arrow indicates a new 1.8-cm aneurysm in the contralateral extracranial internal carotid artery.

Electrocardiography revealed a normal sinus rhythm. Chest radiography indicated a cardiothoracic ratio of 65% (Fig. 1). Computed tomography (CT) showed a true fusiform-type aortic aneurysm with a distal aortic arch diameter of 48 mm, descending aortic diameter of 60 mm, and ascending aortic diameter of 44 mm (Fig. 2). Preoperative 3-dimensional CT (3DCT) showed severe atherosclerotic changes in the aortic arch between the left common carotid and left subclavian arteries and showed that the distal aortic arch aneurysm included the aorta just after the branch of the left subclavian artery. The AKA bifurcated at T10–11. Echocardiographic findings indicated that the Valsalva sinus and aortic annulus had a diameter of 38 and 28 mm, respectively; severe AR and ASD were observed; and the pulmonary blood flow/systemic blood flow ratio was 1.64. ASD was strongly suspected to be type II without a lower margin. The patient exhibited mild tricuspid regurgitation and had a left ventricular ejection fraction of 50% and an estimated right ventricular pressure of 36 mm Hg. Brain 3DCT revealed the clip for the prior cerebral aneurysm and a new 1.8-cm aneurysm in the contralateral extracranial internal carotid artery (Fig. 3).

**Figure 4.**
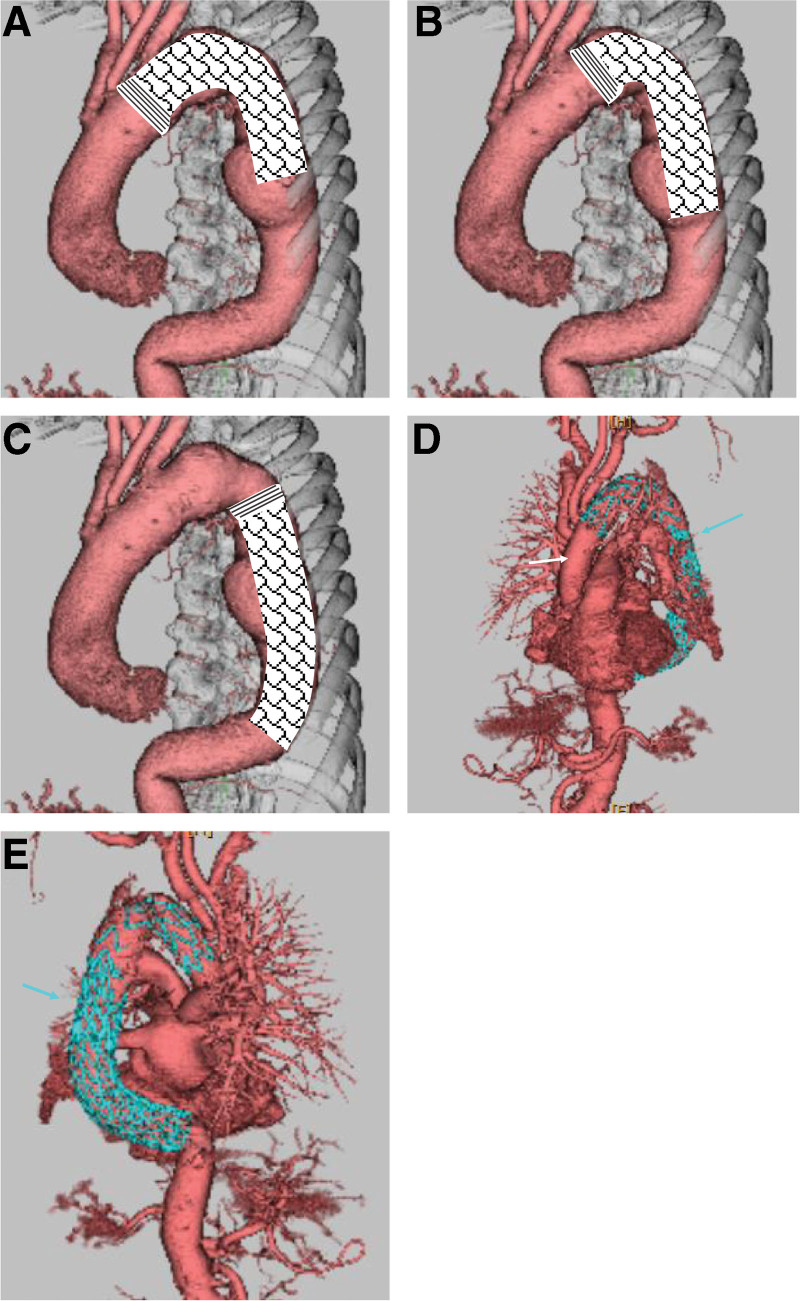
Three-dimensional computed tomography for surgical prediction. (A) Predicted CT with distal anastomosis in zone 2 and insertion of 150 mm of the FET, where the FET does not cover the descending aortic aneurysm. (B) Predicted CT with distal anastomosis in zone 3 and insertion of 150 mm of the FET, where the FET does not cover the descending aortic aneurysm. (C) Predicted CT of a distal aortic arch aneurysm in zone 3 with distal anastomosis on the distal side and insertion of 150 mm of the FET. The FET covers the descending aortic aneurysm but may exceed the Adamkiewicz artery. (A–E) Postoperative 3-dimensional contrast-enhanced CT. The white and blue arrows indicate the graft for total arch replacement and the stent graft for the descending aortic aneurysm, respectively. CT = computed tomography, FET = frozen elephant trunk.

Given the patient’s several preoperative complications and frailty, we considered a single-stage operation using the FET technique (operative risk score [EuroSCORE II]: 12.45%). However, due to the complexity of dialysis and cardiac surgery, we were apprehensive about postoperative hemodynamic instability and considerable bleeding. Additionally, the distal position of the FET was likely to overlap with the AKA bifurcation, which posed a spinal cord protection problem (Fig. 4). Therefore, we chose to perform thoracic endovascular aortic repair (TEVAR) on the descending aortic aneurysm as a second-phase surgery once the patient’s blood pressure stabilized.

The first surgery was conventional sternotomy. After establishing cardiopulmonary bypass, we performed ASD patch closure (2 × 3 cm, GORE-TEX® Cardiovascular Patch; W.L. Gore & Associates, Newark, DE) and aortic valve replacement (27 mm, INSPIRIS; Edwards Lifesciences, Nyon, Switzerland) before the rectal temperature was lowered to 28 °C. The distal aortic arch aneurysm was enlarged to 50 mm, but no adhesions were observed in the surrounding tissues, including the left recurrent nerve. To adequately protect the brain, the target minimum body temperature was adjusted to approximately 28° C. The true aneurysm in the distal aortic arch was resected with selective antegrade cerebral perfusion under whole-body hypothermia. A 7-cm elephant trunk (26 mm, J-graft; Japan Lifeline Co., Ltd., Tokyo, Japan) was inserted, and aortic arch replacement with a 26-mm 4-branched J-graft was performed. Systemic rewarming was initiated after distal anastomosis. We removed the pump without inotropic support, and hemostasis was conducted in the usual manner. The circulatory arrest time, aortic cross-clamp time, and cardiopulmonary bypass time were 50, 154, and 225 minutes, respectively. Overall, the operative time was 5 hours and 50 minutes. To provide sufficient proximal landing for the next TEVAR, we decided to leave the distal 10 cm of the arch replacement graft unbranched.

After the first surgery, the patient experienced atypical pneumonia bilaterally; hence, the second surgery was performed after the pneumonia had improved.

During the second stage, TEVAR was performed, and stent grafts were inserted through the right common femoral artery. We used 2 stent grafts—a Valiant thoracic stent graft with the Captivia delivery system (VAMF 323220; Medtronic Cardiovascular, Santa Rosa, CA) to minimize proximal bird beak occurrence and a C-TAG stent graft with active control system (TGMR 373720; W.L. Gore & Associates) to achieve easier distal alignment. The mean arterial pressure (MAP) was maintained at 60 mm Hg during deployment, with a heart rate of 120 bpm with rapid pacing in the right ventricle. Subsequently, the deployed stent graft was gently dilated using a Reliant balloon (Medtronic Cardiovascular) to remove any pleating from the graft. During deployment, the MAP was maintained at 60 mm Hg, and the heart rate was set at 140 bpm with a pacing lead inserted into the right ventricle to allow the blood pressure to drop during stent expansion.

After confirming stable respiratory function and blood pressure, the patient was extubated in the operating room at the end of surgery. Postoperatively, the patient was discharged without neurological complications or aortic aneurysm enlargement. Postoperative 3DCT showed a good stent position (Fig. 4) and a small type IV endoleak, which disappeared after 3 months.

## 3. Discussion

This report describes the surgical treatment for extensive aortic arch aneurysms in a severely frail patient on hemodialysis. Ideally, the surgical treatment for extensive aortic arch aneurysms should be a minimally invasive, single-stage operation with control of organ ischemia. However, many diseases and anatomical features would be missed if the preoperative examination was neglected. In this case, we identified several pitfalls and ultimately performed 2-stage aortic surgery, achieving good results.

Surgery for extensive aortic aneurysms extending from the aortic arch to the descending aorta is complex and requires careful preoperative planning. Furthermore, conventional open surgery necessitates intraoperative technique selection, circulatory arrest, cerebral protection, and meticulous hemostatic planning. Therefore, it is a complicated operation that cannot be applied to all patients in many institutions. Additionally, an ordinary course, including perioperative management, has not been established. Improvements in surgical techniques, innovations in cardiopulmonary bypass, and modifications in postoperative care plans are paramount to reduce the mortality and sequelae of serious complications associated with these procedures.^[7,8]^

For the repair of the entire arch and descending aorta, the choice of approach is the first consideration. Strategies for repairing complex and extensive aortic diseases include sternal incision, left anterior sternal incision, bilateral anterior sternal incision (clamshell incision), and a 2-stage system with sternal incision and left chest opening (elephant trunk procedure). Deep hypothermic circulatory arrest has also been applied for the protection of brain and abdominal organs, especially the kidneys. Ideally, the procedure should be performed in a single stage and involve extensive aortic repair; nonetheless, there are concerns regarding respiratory complications in elderly and frail patients. Conventional open surgical repair of complex thoracic aortic disease extending from the aortic arch to the descending aorta requires 2 major surgeries: 1 through a mid-thoracic incision and the other via left-sided thoracotomy.^[7,9]^ These procedures are too invasive in high-risk cases, resulting in high operative mortality and serious complications.^[10]^ In 1983, Borst et al^[11]^ introduced the elephant trunk technique to facilitate second-stage surgery. However, the main problem was that the first stage was too invasive, whereas the second stage was delayed, leading to rupture and a complicated second-stage surgery, which did not reduce mortality. As a 2-stage procedure, the elephant trunk technique has several problems:

1. it is technically challenging and requires protection of the recurrent nerve;
2. compression of the elephant trunk due to compression of the false lumen is possible, particularly if the true lumen is small; and
3. a new entry may occur postoperatively and the false lumen may expand rapidly.

Approximately 10% to 15% of patients die between the first and second stages of aortic repair.^[7,12]^ Additionally, some patients cannot undergo the second stage of the procedure because of surgical complications and sequelae of the first stage. In contrast, patients who undergo the second stage of aortic repair have been reported to have almost double the survival rate, which is a critical surgical challenge for improving the distant prognosis.^[13,14]^ Our goal is to achieve the same anatomical repair results as extensive total arch replacement but with fewer surgical techniques and aortic dissection. A surgical approach to complete the extensive repair of the entire aorta in a single step would be ideal.

For our case, we first considered debranching TEVAR because the patient was on maintenance hemodialysis and exhibited muscular frailty. However, as the left subclavian artery could not be coil-occluded because of a shunt in the left arm and the venous vessels around the axillary artery were dilated by the shunt in the radial artery and vein, TEVAR with right and left axillary artery bypass was abandoned. Additionally, preoperative examination revealed aortic insufficiency and ASD (inferior border defect type); hence, we planned to perform extensive total aortic arch repair, aortic valve replacement, and ASD patch closure in a single stage using the midline sternotomy approach. Bypassing the arch vessel from the normal ascending aorta has the advantage of repairing a wide range of aortas at once with off-pump TEVAR. Nevertheless, stroke can occur in 11% of patients,^[15]^ and there are scattered reports of postoperative ascending aortic dissection.^[16]^ While this method is the least invasive, it should not be performed unless there is a solid reason to avoid artificial cardioplegia; minimally, the ascending aorta and neck arteries must be normal.

Based on this history, a method for completing the extensive aortic repair in a single operation, with less dissection area around the aorta, shorter operative time, and less burden on the whole body, is desirable. The FET technique, which has been widely used since the 1990s,^[17]^ has addressed these issues and improved the long-term results. This less invasive method could protect the cerebral blood vessels, coronary arteries, and abdominal organs and prevent pneumonia by preserving the recurrent nerve. The FET technique has been reported to have similar or lower mortality and postoperative stroke rates than the hybrid approach and conventional surgery.^[14,18,19]^ However, the FET technique is associated with a higher risk of spinal cord injury (SCI), is technically more complex, and is more expensive.^[20]^ A meta-analysis conducted by Rezaei et al^[19]^ showed that the pooled prevalence of cerebrovascular accidents, paraplegia, and in-hospital mortality was 7%, 3.5%, and 8.9%, respectively. As the FET technique has become a standard surgical approach, challenges of determining the efficacy of the FET technique and new surgical procedures are being discussed.

Neurological complications such as SCI and stroke are potential problems. Meta-analyses of patients who underwent FET surgery reported a prevalence of 7–8% and 4–7% for cerebrovascular accidents and SCI, respectively.^[19,21]^ Preoperative identification of the AKA is critical; the AKA was identified in 87.6% of cases, was not detected in 10.0% of cases, and branched off from T8–12 in 89.7% of cases.^[22,23]^ Uehara et al^[24]^ reported 1 technique for identifying the location of the AKA using 3DCT before surgery. They accurately measured the distance between the left renal artery and the targeted vital internal costal artery that branches into the AKA using 3DCT. The length from the left renal artery was measured and applied to identify the internal costal artery during the actual surgery

The incidence of SCI after TEVAR is not significantly lower than that after open thoracoabdominal aortic aneurysm repair.^[25]^ However, delayed paralysis/paralysis is the primary manifestation of SCI after TEVAR, as compared with open abdominal surgery.^[26]^ While the pathogenesis of SCI after TEVAR is multifactorial and not fully understood, 2 theories regarding the mechanism of SCI have been suggested: the first relates to inadequate remodeling of the collateral blood network to maintain spinal cord viability, whereas the second refers to atheroembolisation of aortic plaque material into the segmental arteries supplying the spinal cord. Because TEVAR permanently eliminates many segmental arteries occluded by coated thoracic stents, the effect of impaired spinal cord blood flow may continue after the relatively brief aortic cross-clamping during open surgery.^[27,28]^ Feezor et al^[29]^ reviewed 326 TEVAR patients and reported a 30% increase in SCI risk for every 2-cm increase in thoracic aortic coverage.

General guidelines for minimising SCI also include increasing the MAP (i.e., ≥90 mm Hg) and draining the cerebrospinal fluid (≤10 mm Hg) to maintain the spinal perfusion pressure at  ≥ 80 mm Hg. A position paper on spinal cord protection in TEVAR recommends that the blood pressure of patients with SCI should be higher than their preoperative MAP.^[30]^ However, in this case, a low blood pressure had to be maintained because of the presence of a cerebral aneurysm. Furthermore, it was not easy to maintain a high blood pressure postoperatively because of the complicated open-heart surgery for our patient who was on dialysis.

Recently, there has been some debate concerning the value of introducing postoperative spinal fluid drainage in patients presenting with delayed paralysis. In particular, Kakinohana questioned the role of spinal fluid drainage in preventing delayed paralysis after TEVAR and pointed out that its use in this setting is not entirely justified because the mechanism of delayed paralysis is not fully understood.^[26]^ In this patient, spinal fluid drainage could not be performed in the presence of cerebrovascular lesions, and this patient could only be managed with supportive measures.

One option to avoid SCI is to choose a stent graft with a length that does not cover much of the intercostal artery. According to Preventza et al,^[31]^ the risk of spinal cord ischemia is low when the stent length is 100 mm, and stents longer than 150 mm or that extends beyond T8 should be avoided during FET surgery. Reducing the duration of spinal cord and brain ischemia is also a critical consideration. Anastomosis is technically more manageable in zone 2 than in zone 3 and may reduce neurological complications.^[32]^ Recently, there have been reports of anastomosis in zone 0.^[33]^ The main goal is to repair the aorta in a single stage. However, the shorter the stent graft, the higher the rate of secondary aortic intervention (SAI). Aortic events and SAI rates are increased in patients with T10 or less and a FET measuring 150 mm or less.^[3]^ Liebrich et al^[34]^ reported that distal anastomosis in zone 2 with a 100 mm FET led to the treatment of T2–3 and a high rates of SAI.

Preoperative 3DCT revealed true aneurysms in the distal aortic arch and descending aorta and that the AKA branched from T10 to T11. In this case, we first considered total arch replacement with FET because this patient undergoing hemodialysis had multiple diseases and was frail. However, considering that a 100-mm FET was too short and the distal end of a 150-mm FET was too close to the AKA, we decided to perform a 2-stage surgery. As the distal end of the FET could be strictly predicted by 3DCT, 3DCT was used in elective surgery. We believe that confirming the position of the FET with respect to the AKA is essential. In our case, ASD was not an indication for the use of Amplatzer™ Septal Occluder owing to inferior margin defect, and transcatheter aortic valve replacement was not indicated because of pure AR. Due to the location of the aortic aneurysm, the FET was also not indicated. The terrible situation of intraoperative procedure change could have been avoided by performing adequate preoperative examination according to the specific situation of the case.

The strengths of this report are that it is well supported by the literature and that we were able to determine the preoperative anatomy of the patient and provide appropriate treatment. Nonetheless, this report also has some important limitations. First, the patient did not have a well-defined aortic aneurysm prior to the development of the aneurysm, which made it difficult to determine the treatment strategy. Second, we could not clarify the relationship between multiple aortic aneurysms and dialysis or AR.

## 4. Conclusions

Extensive aortic arch surgery using the FET procedure is effective for distal aortic arch and descending aortic aneurysms. Nevertheless, when the position of the AKA is close to the aortic aneurysm and blood pressure control is difficult, a 2-stage procedure and accurate positioning of stent grafts during TEVAR are both desirable.

## Author contributions

All authors contributed equally to the manuscript, critically revised it for intellectual content, read and approved the final version, and agreed to be accountable for this work.

**Investigation:** Akie Shimada.

**Methodology:** Taira Yamamoto.

**Project administration:** Taira Yamamoto.

**Supervision:** Minoru Tabata.

**Validation:** Shizuyuki Dohi, Yasutaka Yokoyama.

**Visualization:** Daisuke Endo, Shizuyuki Dohi, Yasutaka Yokoyama.

**Writing – original draft:** Akie Shimada.

**Writing – review & editing:** Taira Yamamoto.

## Acknowledgment

We would like to thank Editage (www.editage.com) for English language editing.
